# Supplementary material for: Evaluation of the Potential Role of Proprotein Convertase Subtilisin/Kexin Type 9 (PCSK9) in Niemann–Pick Disease, Type C1
Source: Int J Mol Sci. 2020 Mar 31;21(7):2430. doi: 10.3390/ijms21072430 (PMC7178166; doi:10.3390/ijms21072430)
Supplement: Supplementary file 1 [file ijms-21-02430-s001.pdf]

Supplementary Materials:

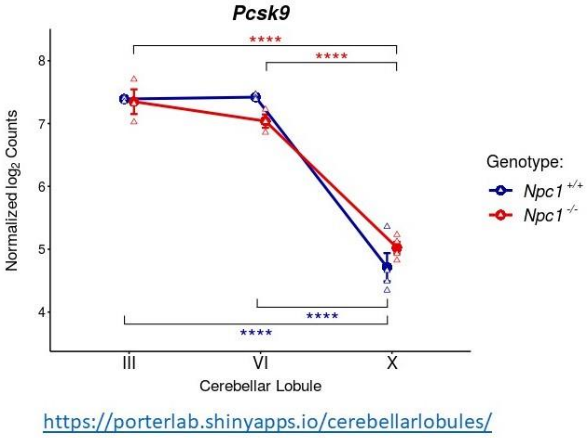

Supplementary Figure 1. RNA-sequencing analysis on the cerebellar lobules of 4.5-week old *Npc1*$^{-/-}$ mice was previously generated by Dr. Kyle Martin [16]. The complete data set may be accessed at https://porterlab.shinyapps.io/cerebellarlobules/. The data identified several pathways and associated genes of interest, including *Pcsk9*. In both *Npc1*$^{+/+}$ and *Npc1*$^{-/-}$ cerebella, *Pcsk9* exhibited an approximately 8-fold increase in the anterior lobules compared to posterior lobule X.

16 Martin, K.B.; Williams, I.M.; Cluzeau, C.V.; Cougnoux, A.; Dale, R.K.; Iben, J.R.; Cawley, N.X.; Wassif, C.A.; Porter, F.D. Identification of Novel Pathways Associated with Patterned Cerebellar Purkinje Neuron Degeneration in Niemann-Pick Disease, Type C1. *Int J Mol Sci* **2019**, *21*, doi:10.3390/ijms21010292.
